# Expression of amygdala mineralocorticoid receptor and glucocorticoid receptor in the single-prolonged stress rats

**DOI:** 10.1186/1471-2202-15-77

**Published:** 2014-06-19

**Authors:** Fang Han, Jinlan Ding, Yuxiu Shi

**Affiliations:** 1 PTSD lab, Department of Histology and Embryology, Institute of pathology and Pathophysiology, China Medical University, Shenyang 110001, China

**Keywords:** Mineralocorticoid receptor, Glucocorticoid receptor, Amygdala, Post-tramumatic stress disorder, Single prolonged stress

## Abstract

**Background:**

Post-traumatic stress disorder (PTSD) is an anxious disorder associated with low levels of corticosterone and enhanced negative feedback of the hypothalamic–pituitary–adrenal (HPA) axis. Previous studies showed that the amygdala not only has an excitatory effect on the HPA axis but also plays a key role in fear-related behaviors. Coticosterone exert actions through binding to the mineralocorticoid (MR) and glucocorticoid receptor (GR), which are abundant in the amygdala. In our previous study, down-regulation of MR and GR in the hippocampus of PTSD rats was found. But the roles of MR and GR in the amygdala of PTSD rats is incompletely understood.

**Results:**

wistar rats were divided into 1 d, 7 d, 14 d groups after single prolonged stress (SPS) and control group. SPS is a reliable animal model of PTSD. Open field test (OF) and elevated plus maze tests (EPM) were performed to examine fear-related behaviors. Morphological changes of the ultrastructure of the amygdala neurons were assessed by transmission electron microscopy (TEM). Dual-immunofluorescence histochemistry was used to determined subcellular distribution and colocalization of MR- and GR-ir. Protein and mRNA of MR and GR was examined by western blotting and RT-PCR. OF and EPM showed enhanced fear in SPS rats. Abnormal neuronal morphology was discovered in the amygdala of SPS rats. The expression of MR- and GR-ir intensity, mRNA and protein within the amygdala decreased after SPS at 1 day, and then gradually recovered by 14 days, although the degree of decrease and recovery were different amongst techniques. We found no change in the MR/GR ratio at 3 levels of the amygdala. But more cytoplasmic distribution and decreased colocalization of MR- and GR-ir were observed in the amygdala after 7 days of SPS.

**Conclusion:**

These data suggest that change of MR and GR in the amygdala are involved in the mechanisms of fear in PTSD.

## Background

Post-traumatic stress disorder (PTSD) is an anxiety disorder that develops after exposure to a life-threatening traumatic experience, and is characterized by intrusive memories, a hyper-arousal state and avoidance of stimuli associated with the trauma [1]. PTSD is also characterized by changes in the neuroendocrine system including abnormal blood corticosteroid concentration and enhanced negative feedback of the hypothalamic–pituitary–adrenal (HPA) axis [2,3]. Corticosteroids inhibit the HPA axis via 2 types of corticosteroid receptors: mineralocorticoid receptors (MR) and glucocorticoid receptor (GR) [3]. Without corticosterone, unbound MR and GR are believed to be primarily localized in the cytoplasm, but can translocate to the nucleus after binding to the hormone ligand. Both receptors show different affinity for corticosteroids; MR has a higher affinity for corticosteroids than the GR [4,5]. At basal levels of corticosterone, MR is believed to be predominant in the maintenance of homeostasis, whereas GR becomes activated when corticosteroid levels increase after stress [3].

Several animal models of PTSD have been developed for mimicking the pathophysiology and behavioral characteristics of PTSD. Among these animal models of PTSD, a single prolonged stress (SPS) model was firstly proposed by Liberzon et al. [6], which consists of 3 stages: 2 h restraint, 20 min forced swim in 37°C water, and exposure to ether anesthesia. SPS rats express enhanced negative feedback of the HPA axis and low levels of corticostone in plasma, which resemble neuroendocrinology changes in PTSD [6].

The amygdala is one of the key regions in the limbic system of the brain and has been thought to have an important role in the emotional memory such as fear. Amygdala has three distinct subgroups: central nucleus, corticomedial nucleus, and basolateral nucleus [7]. The basolateral nucleus is the largest among these three and has been strongly implicated as key sites for stress and fear/anxiety functions [8,9]. Magnetic resource image (MRI) studies reveal significant amygdala volume reduction in adult patients with PTSD [10,11]. Our previous study noted a higher apoptosis rate in the amygdala with TUNEL-staining and double-labeled flow cytometry methods. Changed apoptosis-related protein (Bcl-2 and Bax) were also found in the amygdala of the SPS rats [12]. These studies suggest abnormal amygdala structure and function is involved in PTSD. On the other hand, neuroendocrine studies from Feldman suggest that the amygdala has an excitatory effect on the HPA axis, as noted by increased corticosterone levels after amygdala stimulation, as well as inhibition of the HPA axis responses to stress in rats with amygdala lesions [13,14]. MR and GR have a high density of expression in the amygdala [4]. It has been reported that adding of corticosteroids to the amygdala enhanced anxiety-like behavior [15]. Blockade of both GR and MR produced an increase in startle response [16]. GR and MR antagonist increased the percentage of time the rats spent on open arms, and increased the amount of entries into these open arms in the Elevated Plus-Maze test [17]. These findings suggest that effects of both receptors have been implicated in the fear-enhancing effects of glucocorticoids [18]. The bulk of studies on both receptors have focused on hippocampal neurons, but no work has shed light on their function in the amygdala related to PTSD. In our previous study, down-regulation of MR and GR in the hippocampus of PTSD rats was found [19]. But the roles of MR and GR in the amygdala of PTSD rats is incompletely understood.

In the present study, we aimed to address the effects of SPS on fear using behavioral tests, and determining morphological changes of the amygdala neurons. To explore the expression of MR and GR, we determined the levels of protein and mRNA using dual-fluorescence histochemistry, western blotting and RT-PCR.

## Methods

### Animal model preparation and grouping

A total of 60 male Wistar rats (220–250 g) were randomly divided into 4 groups (15 rats per group): a control group, SPS groups examined on day 1 (1 day), day 7 (7 days) and day 14 (14 days). The control rats remained in their home cages with no handling for 14 days and were killed at the same time as the SPS groups. The SPS rats underwent the SPS procedure on the first day. The SPS procedure was carried out according to the following protocol [6]: a 2 h immobilization (compression with plastic bags), a 20 min forced swim (25°C), a 15 min rest, followed by ether anesthesia (until loss of consciousness). After SPS, the rats were fed routinely. The study was approved by the ethics committee of China Medical University. Experiments were carried out in accordance with the Guidelines laid down by the NIH in the US regarding the care and use of animals for experimental procedures.

### Behavioral test

All rats of each group underwent the behavioral test (the open field (OF) test and elevated plus maze (EPM) test) at two hours before being killed. The EPM test was carried out at one hour after OF test.

#### Open field (OF) test

The open-field test was used to study anxiety/fear-related behavior. The apparatus was surrounded by black walls 40 cm in height, and the floor (100 cm × 100 cm) was divided into 25 squares (20 cm × 20 cm each). During the experiment, each rat was put in the center of the open field, and behavior was recorded for 5 min by an automatic analyzing system. Time of centre cross, the number of centre cross and total cross, and the number of rearing were recorded. The apparatus was cleaned with water using a wet sponge and a paper towel before the introduction of each rat.

#### Elevated Plus Maze (EPM) test

EPM has been well validated in detecting responses to external stressful stimuli. The EPM apparatus consists of a plus-shaped maze elevated above the floor with 2 oppositely positioned closed arms (50 cm × 10 cm), 2 oppositely positioned open arms (50 cm × 10 cm), and a center area (10 × 10 cm). At the beginning, rats were placed in the central area of the maze, facing an enclosed arm. Behavior was recorded with a video camera during the initial 5 min. The number of entries into open arms, into closed arms and the time spent in the open arms, in the closed arms were measured. The percentage of open arm entries (number of entries into the open arm/total number of entries in both arms), and the percentage of time in the open arms (time in the open arms /the time in both arms) were calculated. The measures of anxiety/fear are the percentage (%) of open arm entries and the percentage (%) of time spent on the open arms. The number of closed arm entries is considered locomotor measures.

### Transmission electron microscopy (TEM)

Rats (n = 3) of each group were perfused through the heart with a mixture of 2% paraformaldehyde and 2.5% glutaraldehyde in phosphate buffer. The brains were removed and fixed in the same fixative solution for one night. The amygdala was cut into 1 mm^3^ blocks. The blocks were post-fixed in 1% osmium tetroxide for 2 h at 4°C. After dehydration in graded concentrations of ethanol, the specimens were treated with propylene oxide and embedded in Epon 812 for 2 days at 65°C. Semi-thin sections stained with 1% toluidine blue were examined using a light microscope, and suitable regions were carefully selected for trimming of the blocks. Ultra-thin sections stained with uranyl acetate and lead citrate were examined using a transmission electron microscope (JEM-1200EX; Jeol, Tokyo, Japan).

### Brain tissue preparation and Dual-labeling immunofluorescence of MR and GR in the amygdala

Rats of each group (n = 4) were infused with 300 ml of 0.01 M PBS (pH 7.4) including 4% paraformaldehyde. The whole brains were rapidly removed into the same fixative for 24 h at 4°C. The brains were immersed in 30% sucrose in 0.1 M PB for 3 days for cryosections. The brain tissue was cut into slices of 15 um thickness using a cryostat (Leica CM 3050, Germany). The sections were washed 3 times (5 min each) with 0.01 M PBS, and then treated with 2% BSA in PBS for 2 h at RT for blocking nonspecific reactions. The sections were treated with mouse monoclonal anti-MR antibody (diluted to 1:200; Santa Cruz; CA, USA) and rabbit polyclonal anti-GR antibody (diluted to 1:500; Santa Cruz; CA, USA) in TBS solution for 24 h at 4°C. The sections were washed 3 times with TBS, and then incubated with Alexa Fluor 488 labeled anti-rabbit IgG (1:1,000; Molecular Probes, USA) and Alexa Fluor 546 labeled anti-mouse IgG (1:1,000; Molecular Probes, USA) in 2% BSA-PBS for 2 h at RT. After being washed 3 times with PBS, the sections were observed under a confocal laser-scanning microscope (TCS SP2; Leica, Wetzlar, Germany).

Six slides were randomly selected from each rat. For each slide, 5 randomly selected visual fields in the amygdala were chosen (×40 magnification). We recorded the optical density (OD) of positive cells in each field to evaluate the average OD. The OD of MR and GR-immunopositive cells were analyzed using a Meta Morph/DPIO/BX41 morphology image analysis system.

### Western blotting analysis of MR and GR

Rats (n = 4 per group) were decapitated, and the brains were immediately removed and quick frozen in liquid nitrogen and stored at −80°C. The amygdala was then dissected from brain tissue according to the atlas [20] using a stereomicroscope. The amygdala of each rat was homogenized with a buffer containing 200 mM TBS, 4% SDS, 20% glycerol, and 10% 2-mercaptoethanol, and were denatured by boiling for 5 min. Samples (50 μg/lane) were loaded on a 7.5% SDS-polyacrylamide gel, and electro-blotted onto a PVDF membrane (Millipore Corp., Bedford, MA) from the gel by a semi-dry blotting apparatus (Bio-Rad Laboratories, Inc, Hercules, CA). The PVDF membrane was treated with 1.5% skim milk, 0.05% Tween-20 in TBS (TBST) at 4°C overnight, and then incubated with 1:200 mouse monoclonal antibody against MR (Santa Cruz, USA) or 1:500 rabbit polyclonal antibody against GR (Santa Cruz, USA) at 4°C for 24 h. After being washed 3 times with TBST, the blots were incubated with a second antibody (anti-mouse or anti-rabbit IgG-HRP from Santa Cruz; 1:1,000) for 2 h at room temperature. After incubation, blots were washed 3 times with TBST, and then were visualized using enhanced chemiluminescence (ECL; Amersham Pharmacia Biotech). The same blots were incubated with antibodies against β-actin (Abcam, British; 1:1,000) as positive control. The protein levels of MR and GR were evaluated by calculating the OD ratio of MR/β-actin and GR/β-actin. The OD of MR, GR, and β-actin were analyzed on the Gel Image Analysis System (Tanon 2500R, Shanghai, China). The procedures were repeated 6 times to obtain the average value.

### Total RNA extraction and RT-PCR of MR and GR mRNA

After decapitation, the amygdala from each group (n = 4) was dissected and removed from the brain. Total RNA was extracted using TRIzol (Invitrogen, Japan) according to the manufacturer’s instructions. 1 μg of total RNA was reverse transcribed into cDNA with a RNA PCR Kit (AM Ver.3.0, TaKaRa bio, Otsu, Japan). The primers were synthesized by Shenggong Biotech Limited Company (Shanghai, China). The sequences of MR primers were: 5′-AGA AGA TGC ATC AGT CTGCC-3′ (upper) and 5′-GTG ATG ATC TCC ACC AGC AT-3′ (lower). The sequences of GR primers were: 5′-ATC CCA CAG ACC AAA GCA CCT T-3′ (upper) and 5′-TCC AGT TTT CAG AAC CAA CAGG-3′ (lower). The sequences of β-actin primers were: 5′-GTC ACC CAC ACT GTG CCC ATC-3′ (upper) and 5′-ACA GAG TAC TTG CGC TCA GGA-3′ (lower). PCR was performed using a thermal cycler (XP cycler, Bioer, Japan) consisting of denaturation at 94°C for 5 min, 32 additional cycles at 94°C for 50 s, and then 45 s of primer annealing at 58°C (for MR), 56.5°C (for GR), and 53°C (for β-actin), followed by a final extension at 72°C for 8 min. The lengths of amplified fragments of MR, GR, and β-actin were 380 bp, 540 bp, and 542 bp, respectively. The PCR products were separated after electrophoresis on 1% (w/v) agarose gel, and the optical density of each band was analyzed on the Gel Image Analysis System (Tanon 2500R, Shanghai, China). The levels of MR and GR mRNA were determined by calculating the optical density ratio of MR/β-actin and GR/β-actin.

### Analyzing the percentage of MR- and GR-immunoreactive (ir) cells in the amygdala

Confocal laser-scanning microscopic images (100 μm × 100 μm) from every fifth section in the amygdala were randomly captured using a computer. For each image, approximately 50 cells were counted. The number of MR-ir cells, GR-ir cells, and the cells colocalized with MR- and GR-ir were counted. The percentage of MR-ir cells that showed GR-ir, and that of GR-ir cells that showed MR-ir, were calculated.

### Statistics

The results were expressed as mean ± S.E.M. The differences between normal control group and SPS groups were analyzed by one-way analysis of variance (ANOVA) followed by Tukey’s post-hoc test using SPSS 13.0 software. A level of P < 0.05 was considered to be statistically significant. A level of P < 0.05 was considered to be statistically significant.

## Results

### Animal behavioral test

Effects of SPS on performance in the open field (OF) tests were shown at Table 1. Rats showed a significant reduction in time spent in the central area (*P < 0.05) as well as a reduction in the number of central squares crossed at 1 day, 7 days and 14 days after SPS exposure in comparison with control group. In addition, rats presented a significant reduction in the number of rearings at 1 day, 7 days and 14 days after SPS exposure (*P < 0.05). We did not found the significant difference in the number of total cross among these experimental groups.

**Table 1:** Open-field test

	**Time in centre (s)**	**Number of center cross**	**Number of total cross**	**Number of rearing**
SPS −1 day	12.34 ± 0.54	40.10 ± 3.90	86.58 ± 23.43	18.10 ± 0.89
SPS 1 day	7.76 ± 2.35*	32.65 ± 4.87	75.65 ± 14.30	9.37 ± 1.65*
SPS 7 days	5.13 ± 0.91*	31.35 ± 3.43*	78.69 ± 17.45	6.21 ± 0.79*
SPS 14 days	4.25 ± 1.57*	30.47 ± 2.45*	79.23 ± 9.58	7.47 ± 1.52*

Statistical analysis was carried out by ANOVA test (*P < 0.05 compared with control group).

In the Elevated Plus Maze (EPM) Test (Table 2), the percentage of time in the open arms (time in the open arms /the time in both arms) and the percentage of open arm entries (number of entries into the open arm/total number of entries in both arms) were calculated. Rats showed a significant reduction in the percentage of time spent in the open arm (*P < 0.05) and percentage of the number of entries into open arms (*P < 0.05) at 1 day, 7 day and 14 day after SPS exposure in comparison with control group. There were no difference in the number of close arm entries among control group and SPS groups. These results indicated SPS induced increased fear/anxious-related behaviors.

**Table 2:** EPM test

	**Time spent in open arm (%)**	**Number of open arm entries (%)**	**Number of close arm entries**
SPS −1 day	18.34 ± 3.21	37.57 ± 2.37	13.51 ± 6.21
SPS 1 day	13.93 ± 2.57	25.64 ± 1.94*	9.46 ± 4.46
SPS 7 days	12.52 ± 2.15*	20.37 ± 2.18*	12.13 ± 3.85
SPS 14 days	11.28 ± 1.09*	21.55 ± 2.35*	10.43 ± 2.38

Statistical analysis was carried out by ANOVA test (*P < 0.05 compared with control group).

### TEM analysis of the morphological changes in the amygdala cells of SPS rats

**Figure 1:**
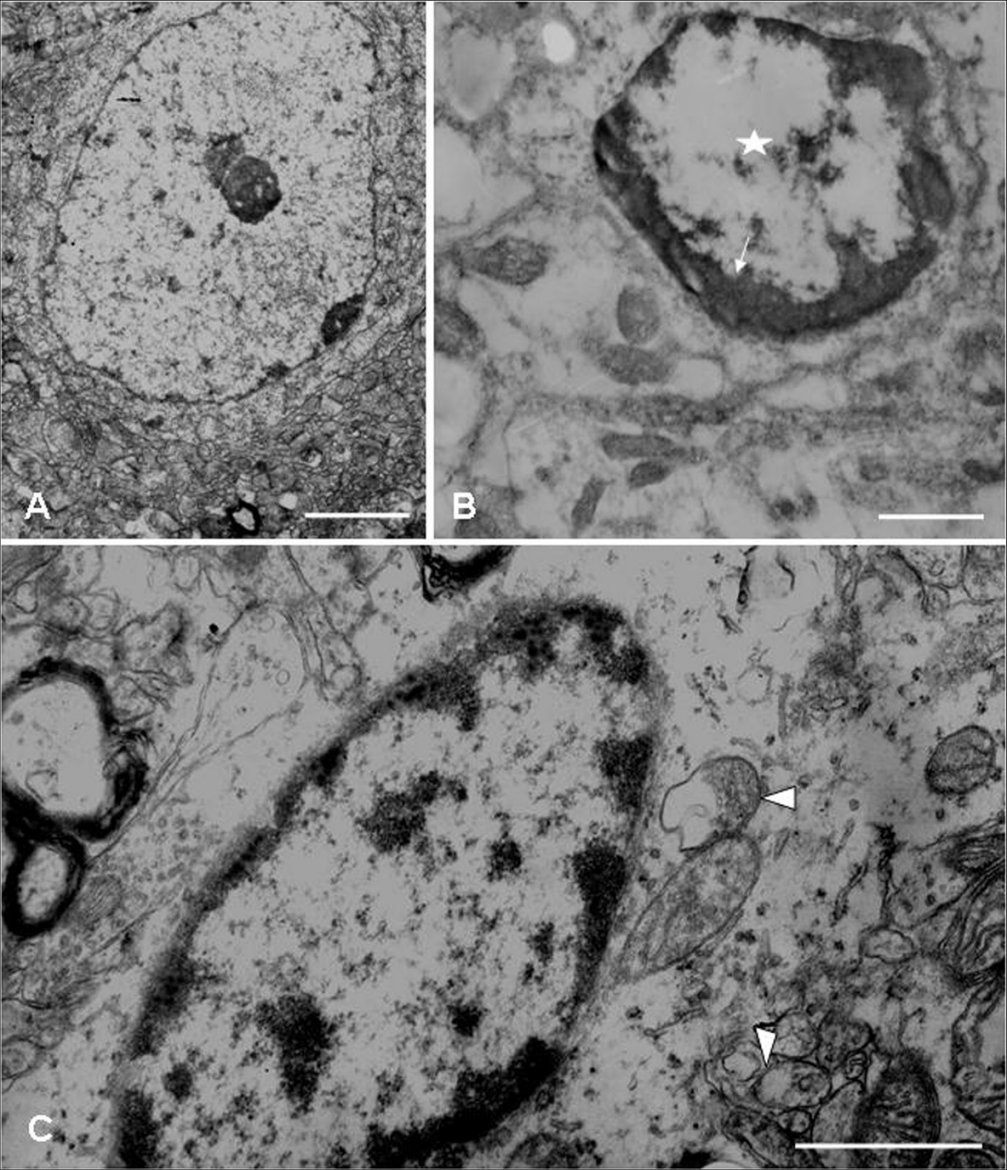
**TEM images of the amygdala in SPS rats.** TEM images of the amygdala. **A**: Normal control amygdala neuron, displaying abundant organelles such as mitochondria, endoplasmic reticulum, and ribosomes. The nucleus is large and round, density of chromatin is uniform, and nucleolus is clear. **B**: Chromatin condensation (shown with arrow) and nucleolus disappearance (shown with star) is shown in the amygdala cells of SPS 7 day rats. **C**: The mitochondria showed swelling, vacuolation and loss of crest (shown with arrowhead) in cells of the amygdala from SPS 7 day rats. Scale bar, 300 nm.

As shown in Figure 1, the amygdala cells in the control rats exhibited normal morphology (Figure 1A). Some cells in the amygdala of SPS 7 day rats exhibited abnormal morphology including chromatin condensation (shown with arrow, Figure 1B) as well as nucleus fragmentation (shown with asterisk, Figure 1B). Also, some cell organelles showed abnormal morphology, such as the mitochondria expressed swelling, vacuolation (shown with arrow head, Figure 1C). These change were also found in the amygdalar cells of SPS 1 day and 7 day rats (data not shown). These changes in cell morphology and cell organelles suggested that SPS induced damage of the amygdala cells.

### Coexpression of MR- and GR-ir in the amygdala

**Figure 2:**
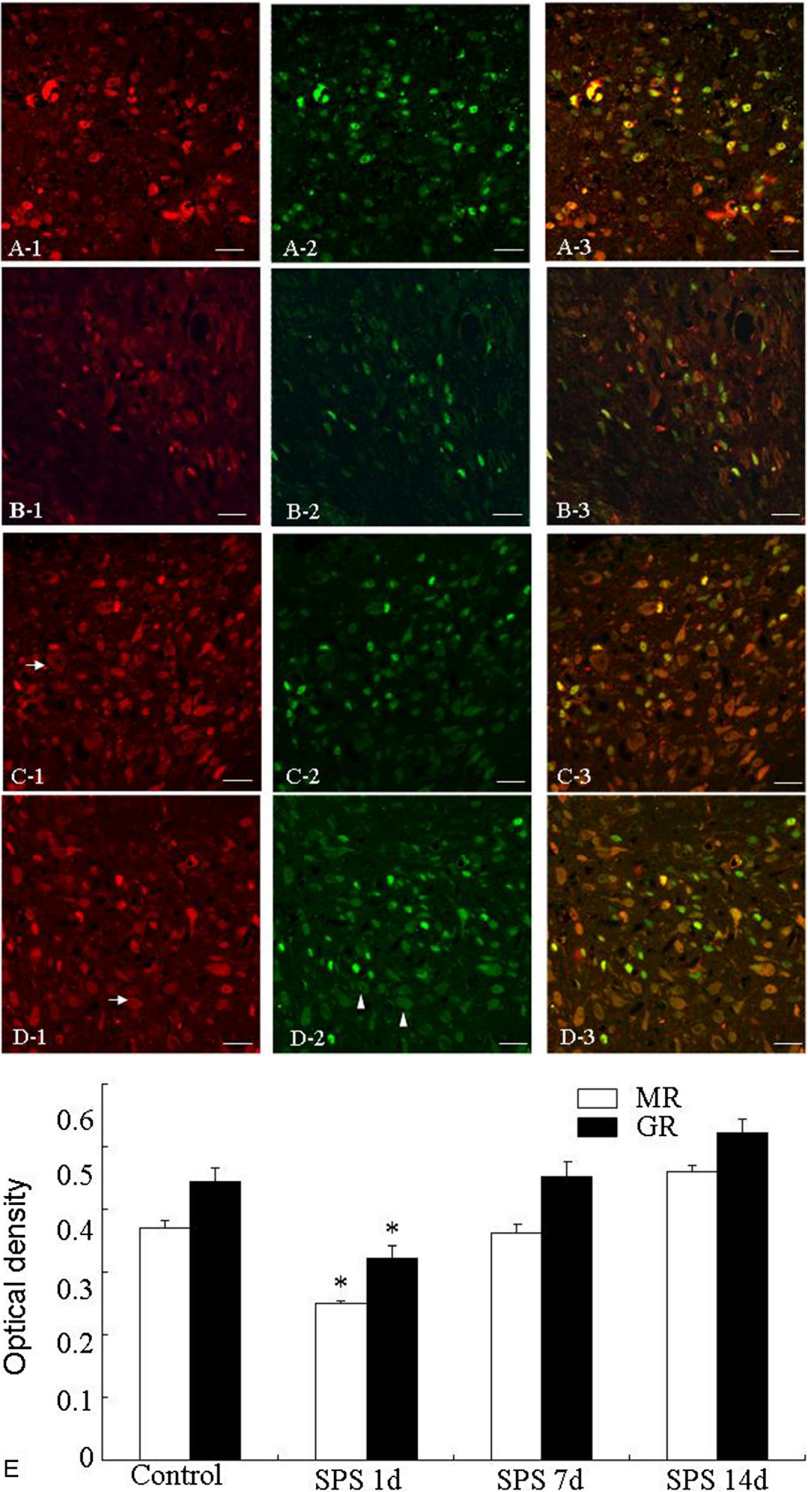
**Effects of SPS on the intensity and localization of MR- and GR-ir in the amygdala.** Dual-immunofluorescent images for the MR- and GR-ir in the amygdala. Images of MR-ir (red; left panel), GR-ir (green; middle panel), and colocalization of MR- and GR-ir (yellow; right panel) in the control group **(A)**, 1 day SPS **(B)**, 7 days SPS **(C)**, and 14 days SPS **(D)**. **E** shows quantitative analysis of MR and GR intensity using immunohistochemistry. The intensity of MR- and GR-ir decreased in the 1 day SPS, and remained virtually unchanged in the 7 days, 14 day SPS in comparison with control group. MR- (arrow) and GR-ir (arrow head) showed more cytoplasmic distribution in the amygdala of the 7 days and 14 days SPS. *P < 0.05 vs. the control group.

**Figure 3:**
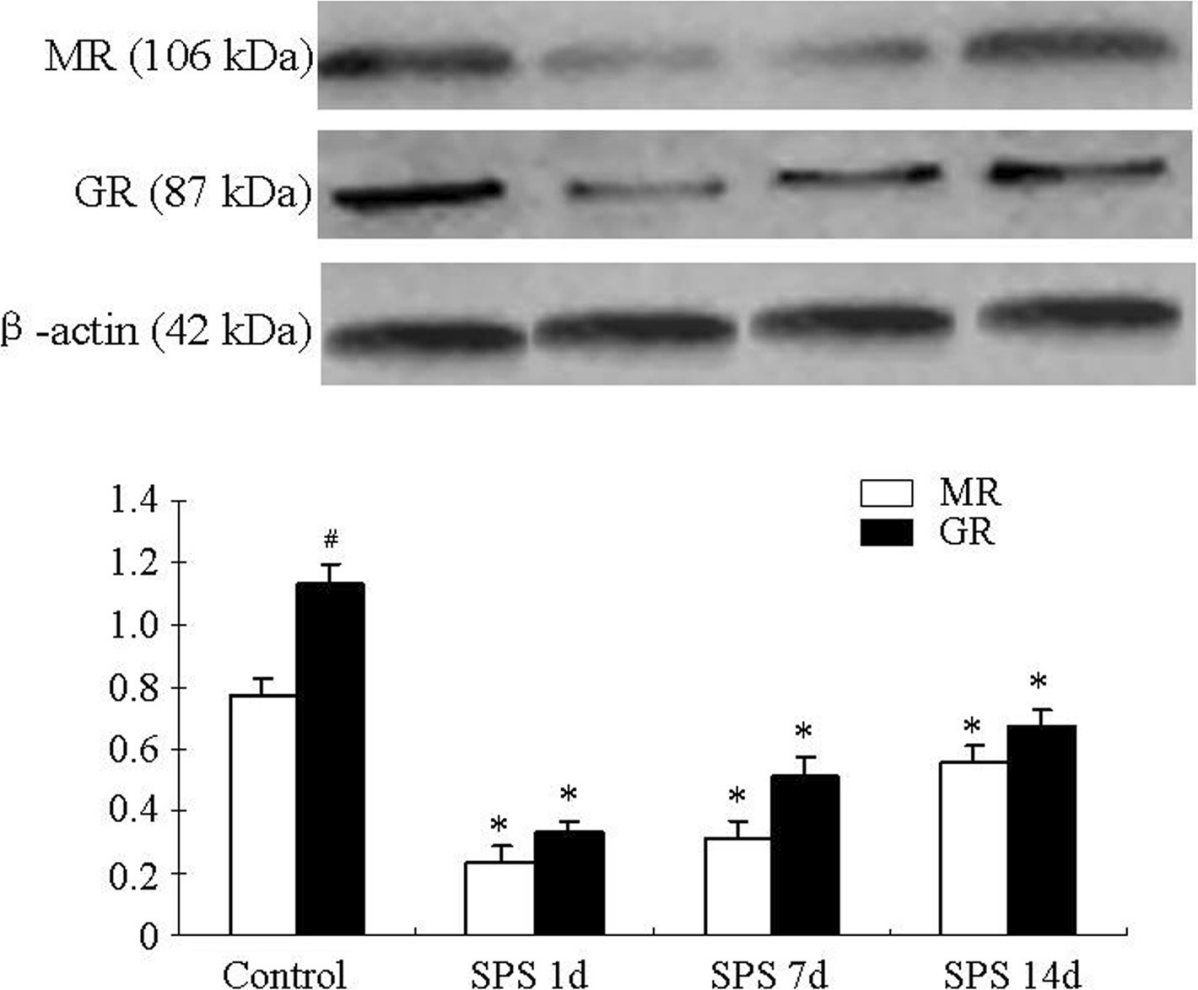
**Western blot of MR and GR in the amygdala of SPS rats.** Figure 3 shows that MR and GR protein expression in the amygdala and quantitative analysis based on western blot results. A significant decrease in MR and GR protein expression in the amygdala was observed at 1 day, 7 days and 14 days after SPS exposure in comparison with the control group. But MR and GR protein showed an increased tendency at 7 and 14 days in comparison with 1 day after SPS (no significant difference). In the control group, MR protein was significantly higher than GR protein. *P < 0.05 vs. the control group; #P < 0.05 vs. MR.

Double immunofluorescent staining clearly showed that MR- and GR-ir was observed in the cytoplasm and nucleus of the amygdala cells (Figure 2A), but MR-ir (Figure 2A-1) showed more cytoplasmic distribution than GR-ir (Figure 2A-2) in the control rats. Merged yellow fluorescence confirmed the colocalization of MR- and GR-ir in the nuclear, but not cytoplasmic, compartment of the amygdala cells (Figure 2A-3). The effect of SPS on the distribution and intensity of MR- and GR-ir were examined. At 1 day after SPS (Figure 2B), the intensity of MR- (Figure 2B-1) and GR-ir (Figure 2B-2) within the amygdala significantly decreased in comparison with the control group. At 7 days (Figure 2C) and 14 days (Figure 2D) after SPS, MR- and GR-ir showed no significant difference in intensity in comparison with control group. But, SPS induced more cytoplasmic distribution of MR- ir and GR-ir in the SPS 7 days and 14 days.

We counted the number of colocalized MR- and GR-ir cells, and found that 93.5 ± 2.3% MR-ir cells and 92.8 ± 3.4% GR-ir cells expressed GR- and MR-ir in the amygdala of the control group, respectively. In the SPS 1 day rats, 34.3 ± 2.4% MR-ir cells expressed GR-ir and 76.5 ± 4.1% GR-ir cells expressed MR-ir. In the SPS 7 day rats, 38.7 ± 2.9% MR-ir cells expressed GR-ir and 85.2 ± 3.2% GR-ir cells expressed MR-ir, respectively. In the SPS 14 day rats, MR-ir cells showed 91.4 ± 2.45% GR-ir and 69.3 ± 2.7% GR-ir cells showed MR-ir, respectively (Table 3).

**Table 3:** The percentage of cells expressing MR- or GR-ir in the amygdala

	**MR-ir cells with GR-ir (%)**	**GR-ir cells with MR-ir (%)**
Control	93.5 ± 2.3%	92.8 ± 3.4%
SPS-1 day	34.3 ± 2.4%	76.5 ± 4.1%
SPS-7 day	38.7 ± 2.9%	85.2 ± 3.2%
SPS-14 days	91.4 ± 2.45%	69.3 ± 2.7%

### Western blotting of MR and GR in the amygdala

The protein signals for MR, GR, and β-actin appeared on Western blot at 106 KD, 87 KD, and 42 KD, respectively. Changes in MR and GR expression within the amygdala region between control and SPS rats are presented in Figure 3. The band mean density of the control group was set as 100%. Data were expressed as normalized optical density. β-actin protein level was used as the internal control at each time point. MR and GR expression were significantly decreased in SPS 1 day, 7 days and 14 days rats in comparison with control rats (Figure 3).

### RT-PCR of MR mRNA and GR mRNA

**Figure 4:**
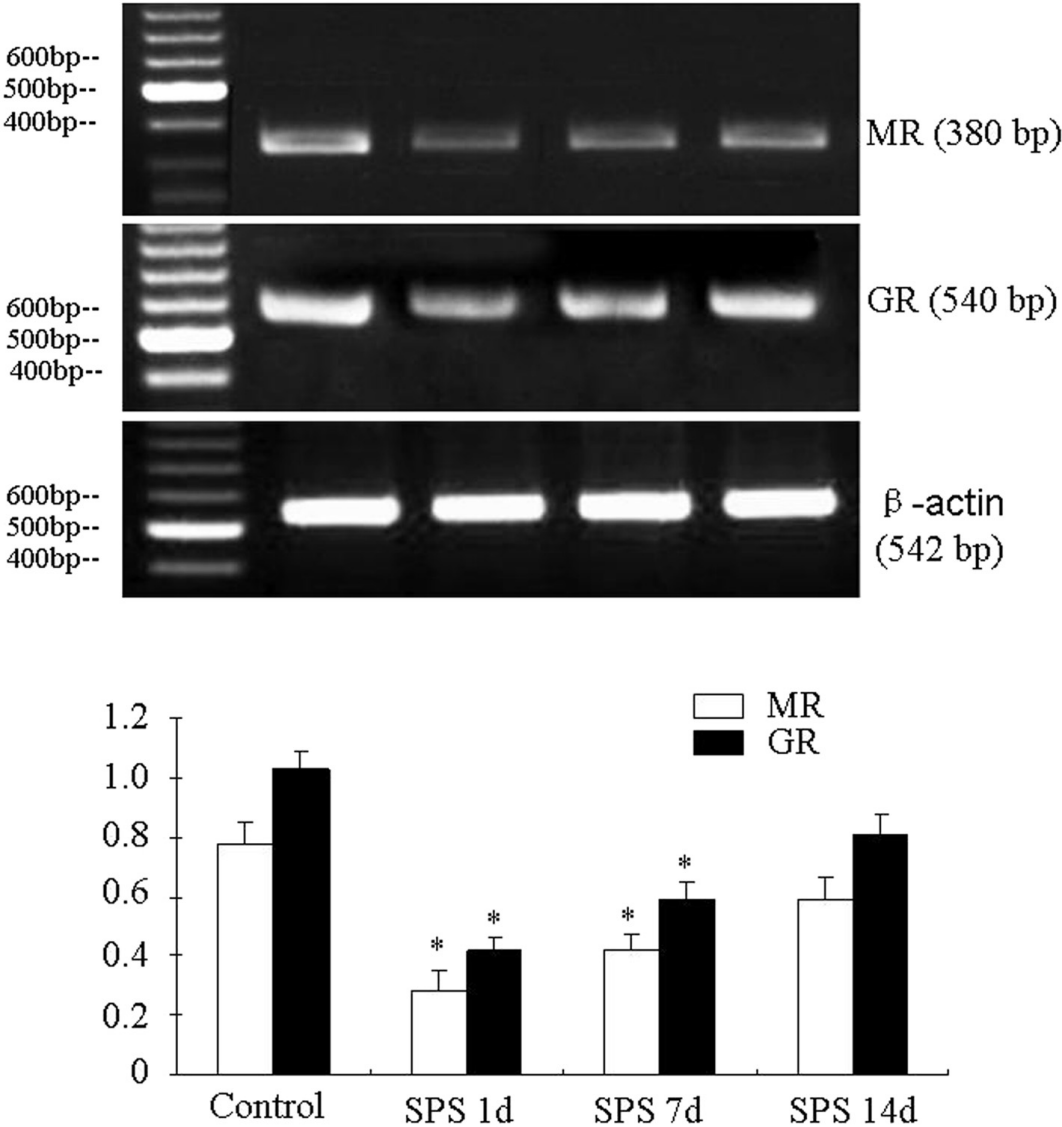
**RT-PCR of MR and GR in the amygdala of the SPS rats.** MR and GR mRNA expression and results from quantitative analysis. MR and GR mRNA level in the amygdala of SPS 1 day and 7 days were lower than that in the amygdala of control rats. But no significant difference was found between control group and SPS 14 days group. *P < 0.05 vs. the control group.

The levels of MR and GR mRNA were normalized with β-actin mRNA levels. Similar to the Western blot, the mRNA levels of MR and GR in the SPS rats were significantly decreased on day 1, day 7 and then recovered at 14 days, compared to the control group (Figure 4).

### Ratio of MR/GR in the amygdala

**Figure 5:**
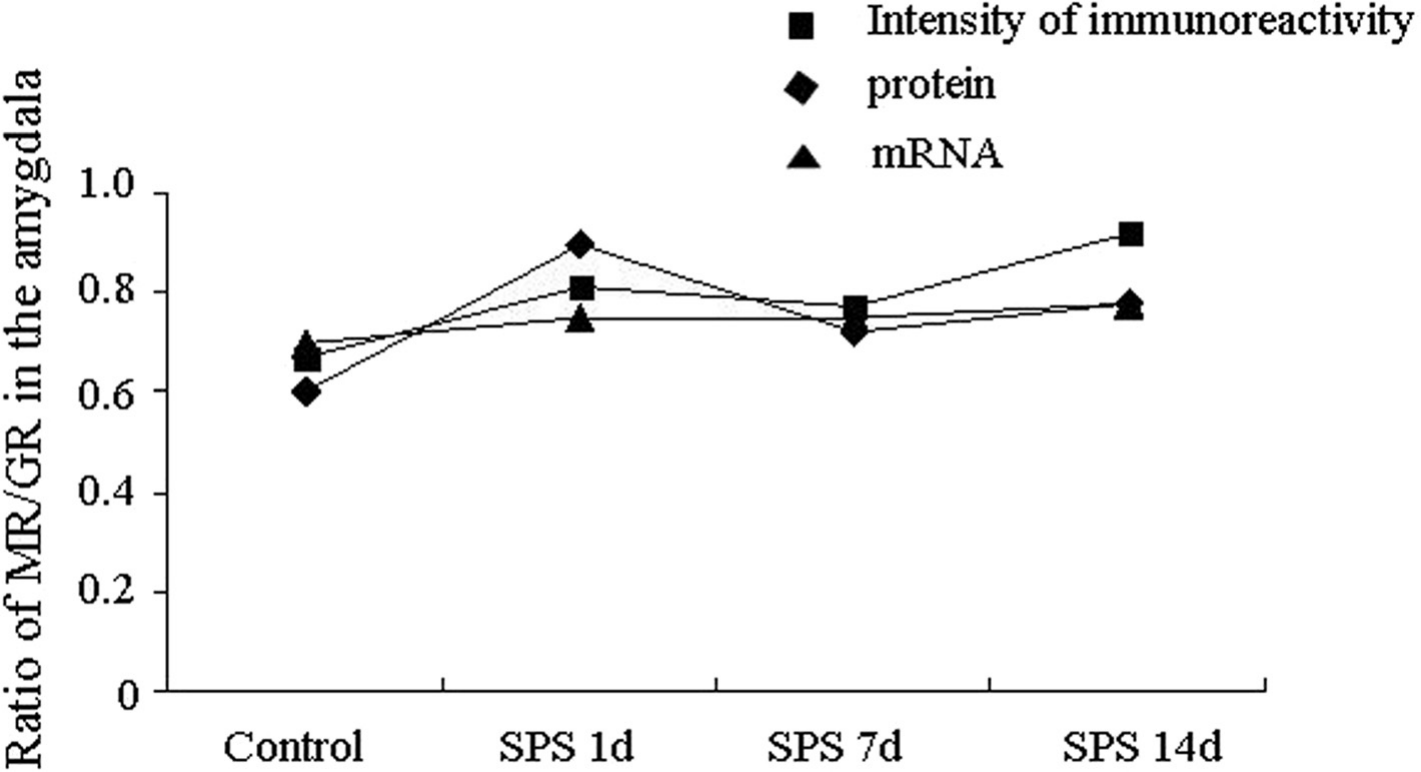
**MR:GR ratio in the amygdala cells.** MR:GR ratio of protein and mRNA expression shows no significant change between groups.

We compared the ratio of MR/GR in the amygdala at intensity of immunoreactivity, protein and mRNA expression among the control group, 1, 7, and 14 days after SPS. The ratio of MR/GR at 3 levels showed no significant change at 1, 7, and 14 days after SPS in comparison with the control group (Figure 5).

## Discussion

The amygdala plays an important role in fear learning and fear expression [21]. MRI studies reveal different results on the volume of the amygdala in patients with PTSD: smaller and unchanged amygdala volumes in patients with PTSD [22,23]. In vivo proton magnetic resonance spectroscopy (^1^H-MRS) revealed the high NAA/Cr and Cho/Cr ratios, which reflect a hyperresponsive amygdala [10,11]. Increased anxiety-like behavior may also be an important symptom cluster in patients with PTSD. Our study found that SPS induced distinct, enhanced fear because SPS decreased the number of entries into the open arms in the EPM test, and locomotor activity within the inner parts of the field in the OF test. These results are consistent with previous studies [24,25]. Our TEM observation also found that SPS induced abnormal morphological changes in the cells of the amygdala: swelling mitochondria, vacuolation and loss of crest in the mitochondria, existence of chromatin condensation, nucleus fragmentation and nucleolus disappearance, which reflected damage of the amygdala neurons after SPS stimulating.

It is known that MR maintains the basal activity of the HPA axis, so MR plays a lesser role in regulating the HPA axis during stress [3,26]. The hippocampal MR in contrast has neuron protection function [26]. Down-regulation of hippocampal MR in PTSD is associated with hippocampal tissue loss [19]. Except for the hippocampus, our previous studies using SPS rats examined neuronal loss in the amygdala [12,24]. We speculated that low expression levels of MR in the amygdala promoted the loss of neurons, leading to a dysfunctional amygdala in PTSD. Meanwhile, decreased MR function is implicated in increased fear-related behaviors in animals [27]. So a decrease in amygdala MR in PTSD could be associated with fear-related clinical phenomena, including startle-response, hyper-vigilance, and increased anxiety. On the other hand, decreased expression levels of amygdala GR in the present study was observed, but the degree of expression change in GR is less than that of MR. The excitatory effects of the amygdala on the HPA axis responses are mediated by hypothalamic serotonin [15]. Our pervious study examined activity of hypothalamic 5-HT1A receptors in the alterations of GR in the hippocampus, and CRF in the hypothalamus of SPS rats [25]. GR signaling in the amygdala suppresses excitatory transmission to the HPA axis [28]. So we speculate that decreased amygdala GR may be involved in hyperactivity of the HPA axis in PTSD. The present study found that SPS causes a decrease in the expression of MR and GR in the amygdala of SPS rats at the level of protein and mRNA. But other studies using Sprague–Dawley rats show that amygdala GR did not change after SPS [29,30]. The different results may depend on the strain of rat and differences in GR and MR expression. In general, decreased MR and GR promote neuronal death in the amygdala, causing changes in amygdala volume and enhanced fear in PTSD.

The present study revealed that MR-ir shows a greater distribution in the cytoplasm compared to GR-ir, and SPS caused more cytoplasmic distribution of both receptors. It is known that MR and GR are hormone-dependent transcription factors, translocating to the nucleus after binding to the hormone ligand, and then regulating neuronal excitability, growth and cell survival and neuroendocrine function [31]. We believe that an increase in cytoplasmic distribution of MR and GR after 7 days SPS indicates that cytoplasmic MR and GR could not regulate neuronal activity, because low glucocorticoids or changes in the structures of MR and GR protein induced by low glucocorticoids [32]. This may explain why, after 7 days SPS, increased levels of MR and GR protein and mRNA could not correct dysfunctions of the amygdala. On the other hand, the present study clearly showed that MR- and GR-ir was colocalized in the amygdala. Colocalization of MR- and GR-ir in the hippocampus has been reported [31,33]. Studies from in vitro and in vivo experiments suggest that MR and GR form not only homodimers but also heterodimers [33,34]. Colocalized cells could be explained by the existence of MR and GR heterodimer. Receptor heterodimers may contribute to the biphasic excitation of neurons to corticosterone [3,26]. Decreased numbers of MR/GR colocalized cells after SPS suggests a decrease in neuronal activation in response to corticosterone in PTSD. In sum, the decrease in the numbers of colocalized cells as well as increased cytoplasmic distribution of both receptors in the amygdala of SPS rats provide evidence for decreased function of MR and GR in the amygdala of SPS rats.

The balance of MR and GR expression in the brain plays a key role in the regulation of neuronal excitability, stress responses and behavior [3,26]. The normal expression ratio of these receptors is considered a protective factor against responses to stress, and promotes health, homeostasis, and adaptation [3]. A change in balance of both receptors alters the ability to maintain homeostasis, which in turn alters neuronal excitability, stress responsiveness, and behavioral adaptation to a condition of enhanced vulnerability to disease [26]. An imbalance in the MR:GR ratio in the hippocampus of SPS rats has been described in our previous study [19]. In contrast to the hippocampus, we did not detect a significant difference in the MR:GR ratio in the amygdala of SPS rats. However we cannot rule out the possibility of functional actions influencing neurons in the PTSD amygdala through mechanisms mediated by the MR:GR ratio. The MR:GR ratio in the amygdala following SPS was quite different from that in the hippocampus. Differential effects of stress on the hippocampus and the amygdala have been described [35]. One possible explanation is that the MR:GR ratio and regulation might vary by brain region, e.g., in low birth weight related depression, there is a change in the MR:GR ratio in the hippocampus but not in the amygdala [31,36]. Sarabdjitsingh has also reported that stress and corticosterone change synaptic potentiation in the amygdala in a manner opposite to that seen in the hippocampus [37]. These studies have delineated region-specific effects of corticosterone in the neuronal physiology between the hippocampus and the amygdala. Pervious studies also indicate that the action of MR/GR in the amygdala may be more unique or flexible [33,38,39].

## Conclusion

Our study examined that SPS induced enhanced fear/anxious. TEM revealed abnormal morphology of the amygdala neurons. The expression of MR- and GR-ir intensity, mRNA and protein within the amygdala decreased after SPS at 1 day, and then gradually recovered by 14 days, although the degree of decrease and recovery were different amongst techniques. But the MR:GR ratio did not show significant change. Dual fluorescence histochemistry showed increased cytoplasmic distribution of MR- and GR-ir in the SPS group compared to the control group. These data indicate that activation of MR and GR in the amygdala are involved in the mechanisms of fear in PTSD.

## Competing interests

The authors declare no competing interests.

## Authors’ contributions

For regarding of author’s contributions, YS and FH proposed and designed this study. FH executed the experiment including behavious test, transmission electron microscopy, double-immunofluorescence histochemistry, RT-PCR. JD executed western blotting and RT-PCR; FH conducted analysis of data and wrote the manuscript. All authors read and approved the final manuscript.
